# ALKBH5 Promotes Multiple Myeloma Tumorigenicity through inducing m^6^A-demethylation of SAV1 mRNA and Myeloma Stem Cell Phenotype

**DOI:** 10.7150/ijbs.64943

**Published:** 2022-03-06

**Authors:** Tingting Yu, Lan Yao, Hua Yin, Yao Teng, Mei Hong, Qiuling Wu

**Affiliations:** Institute of Hematology, Union Hospital, Tongji Medical College, Huazhong University of Science and Technology, Wuhan, China

**Keywords:** multiple myeloma, m^6^A, ALKBH5, Cell proliferation, Apoptosis, Hippo pathway, multiple myeloma stem cell

## Abstract

N6-methyladenosine (m^6^A) is the most prevalent modification to RNA in higher eukaryotes. ALKBH5 is an RNA demethylase that impacts RNA export and metabolism, and its aberrant expression is associated with the generation of tumours. In this study, we found that ALKBH5 was highly expressed in both primary CD138^+^ plasma cells isolated from multiple myeloma (MM) patients and MM cell lines. Downregulation of ALKBH5 inhibited myeloma cell proliferation, neovascularization, invasion and migration ability, and promoted the apoptosis *in vivo and in vitro*. MeRIP-seq identified the SAV1 gene as main target gene of ALKBH5. Inhibiting ALKBH5 in MM cells increased SAV1 m^6^A levels, decreased SAV1 mRNA stability and expression, suppressed the stem cell related HIPPO-pathway signalling and ultimately activates the downstream effector YAP, exerting an anti-myeloma effect. Additionally, MM stem cell phenotype was suppressed in ALKBH5-deficient cells and the expression of pluripotency factors NANOG, SOX2 and OCT4 were also decreased. Altogether, our results suggest that ALKBH5 acts as an oncogene in MM and might serve as an attractive potential biomarker and therapeutic target.

## Introduction

Multiple myeloma (MM) is a malignant plasma cell disease characterized by multifocal proliferation of clonal, long-lived plasma cells and monoclonal immunoglobulin production, usually accompanied by anaemia, bone destruction, hypercalcaemia and renal dysfunction and damage to organs.^1^ There has been tremendous progress in the treatment of MM in recent years. The application of a new generation of drugs including proteasome inhibitors (PIs), immunomodulatory drugs, monoclonal antibody and histone deacetylase inhibitors has significantly extended the overall survival of MM patients. However, MM is still an incurable disease.^2^ Exploring new forms of pathogenesis and developing novel therapeutic strategies are vital to further improve the survival and prognosis of patients with MM.

N6-methyladenosine (m^6^A) is an abundant internal chemical modification of messenger RNAs (mRNAs), microRNAs and long noncoding RNAs in eukaryotic cells.^3^, ^4^ It plays vital roles in numerous RNA metabolic processes, including RNA splicing, processing, nuclear transport and mRNA degradation.^5^, ^6^ M^6^A is a reversible modification dynamically maintained by a methyltransferase complex and demethylases.^7^, ^8^ Two well-known demethylases, alkylation repair homologue protein 5 (ALKBH5) and fat mass and obesity-associated protein (FTO), both in the AlKB family of dioxygenase, eliminate the m^6^A modification from RNA facilitated by Fe^2+^ and α-ketoglutaric acid (α- KG).^9^ ALKBH5 plays a critical role in mRNA synthesis in the nucleus and the output of mRNA from the nucleus, thus resulting in a significant decrease in cytoplasmic RNA levels.^10^ ALKBH5 has been demonstrated to be involved in the tumorigenesis of several kinds of cancers. It accelerates the proliferation, invasion and metastasis capacity of pancreatic ductal adenocarcinoma cells by activating the Wnt signalling pathway.^11^ In addition, ALKBH5 increases the level of NANOG mRNA in breast cancer cells and promotes the enrichment of breast cancer stem cells.^12^ What's more, ALKBH5 promotes tumorigenesis in acute myeloid leukemia (AML) via facilitating leukaemia stem/initiating cells (LSCs/LICs) 13, 14 However, the role of ALKBH5 in MM has rarely been reported.

The HIPPO pathway is an evolutionally conserved signalling pathway that plays a crucial role in mediating organ size control, tissue regeneration and stem cell self-renewal.^15^ The core Hippo components include mammalian sterile 20-like 1/2 (MST1/2), Large tumour suppressor homologue 1/2 (LATS1/2), Salvador (SAV1), MOB kinase activator 1A/B (MOB1 A/B), and Yes-associated protein (YAP)/transcriptional coactivator with PDZ-binding motif (TAZ).^16^ Recent studies have highlighted the vital role of the Hippo pathway in the occurrence and development of a variety of tumours. Knocking out the SAV1 gene induced hepatocellular carcinoma in mice via the activation of YAP/TAZ,^17^ and high YAP/TAZ levels were closely associated with the poor prognosis of breast cancer, gastrointestinal cancer, and non-small cell lung cancer.^18^-^20^ Recently, it was found that increased YAP significantly promotes the apoptosis of myeloma cells.^21^ However, the specific upstream regulatory mechanisms of the Hippo pathway remain unclear.

In this study, we reveal that MM cells have increased expression of ALKBH5, which is positively correlated with MM advanced stage. Moreover, we investigated its underlying molecular mechanisms and found that ALKBH5 knockout in MM cells inhibits proliferation, promotes apoptosis and regulates MM stem cell phenotype by enhancing the m^6^A level of SAV1 and inhibiting the HIPPO pathway.

## Materials and Methods

### Culture of MM primary cells and cell lines

Mononuclear cells were isolated from bone marrow samples of newly diagnosed MM patients and healthy volunteers by Ficoll at Union Hospital, Wuhan, China (Haoyang, Tianjin, China). The present study was approved by the institutional ethics committee of Huazhong University of Science and Technology. CD138^+^ plasma cells were selected with CD138^+^ microbeads (Miltenyi, Auburn, CA, USA). RPMI8226, U266, ARH-77 and NCI-H929 MM cell lines were purchased from American Type Culture Collection (ATCC) (ATCC, Manassas, VA, USA) and cultured in complete RPMI 1640 medium (Gibco, Grand Island, NY, USA) supplemented with 10-15% FBS (Natocor, Villa Carlos Paz; Argentina) in 5% CO_2_ at 37°C.

### Lentivirus transfection

A total of 1×10^5^ cells were placed in the 24-well plate, and then, 20 μl of polybrene and virus solution (GeneChem, Shanghai, China) was added to wells with sufficient mixing, and the cells were cultured in 5% CO2 at 37°C. Twelve to sixteen hours later, the medium was changed, and the cells continued to be cultured under the same conditions. Stable ALKBH5-knockdown and ALKBH5-overexpressing cells were obtained after being screened by puromycin (Sigma, St. Louis, MO, USA).

### Transwell assays

Cell migration and invasion ability was analyzed by the Transwell assay.

**Migration ability test:** 50,000 cells (200µl) were placed in the Transwell chambers (8µm, Corning, USA), and 600µl RPMI-1640 medium containing 25% FBS was added to the lower compartment. 24h later, the cells in the upper chamber were wiped away. Cells on the lower surface of the chambers were fixed and stained and then counted under an optical microscope (magnification, ×400).

**Invasive ability test:** Matrigel matrix (Corning, NY, USA) was mixed with serun-free medium in a 1:8 ratio. The diluted mixture was applied on the underside of the Transwell chamber at 100 µl/well in a 37°C incubator for 4 h, and the other steps were the same as the migration experiment.

### RIP-qPCR

RIP-qPCR was performed as described with minor modifications.^22^ 5 μg ALKBH5 and IgG control antibodies were used for RIP assay. Co-precipitated RNAs were then determined by RT-qPCR.

### ELISA

Myeloma cells were cultured in complete RPMI 1640 medium, 24h later, the supernatant was collected to measure the levels of VEGF using ELISA kit according to the manufacturer's instruction (Multisciences biotech, Hangzhou, China).

### Side population cell detection

A side population (SP) assay was conducted using Hoechst staining as previously described.^23^ A total of 1×10^6^ MM cells were sustained in 1 mL of RPMI1640 medium supplemented with 0.5 μL of Hoechst dye (Sigma, St Louis, MO, USA) for 90 min at 37℃ either alone or in the presence of 50 mol/L verapamil. Then, the cells were washed with cold PBS to terminate the reaction. The excitation wavelength was set to 355 nm, and the detection wavelengths were 450 and 675 nm. Cells negative for Hoechst red and Hoechst blue stain were considered positive for side population cells.

### Flow cytometry

Cells were washed with PBS and then resuspended in binding buffer at a density of 1 × 10^6^ cells/ml. 100 μl of cell suspension was transferred to a 5 ml culture tube, next, 5μl CD138-BV421 and CD34-APC antibody (BD Bioscience, San Diego, CA, USA) were added to the tube. The mixture was vortexed and incubated for 15 min at 4°C in the dark. Finally, the samples were analysed by flow cytometry(BD Bioscience, San Diego, CA, USA).

### Stem cell sphere formation assay

ALKBH5- and control RPMI8226 cells were plated in 6-well plates and cultured in prepared stem cell medium. Briefly, stem cell medium was prepared by RPMI 1640 medium supplemented with rh stem cell factor, rh GM-CSF, rh IL-3 and EPO (Biolegend, San Diego, CA, USA) and used immediately after preparation. The cell spheroids larger than 50μm in diameter under the microscope was counted as stem cell spheres. Sphere formation ability was determined by the number of stem cell spheroids that formed 14 days after seeding.

### MeRIP-sequencing and gene-specific m^6^A qPCR

The purified RNA samples were cut into 100 bp fragments according to a previous study^22^. Then, RNA samples were incubated with m^6^A primary antibody (Abcam, Cambridge, UK) and immunoprecipitated with protein A beads (Merck Millipore, Billerica, MA, USA) for 2 h at 4 °C. Captured RNA was washed and purified by an RNA Clean and Concentrator kit (ZYMO Research, Beijing, China). Sequencing was carried out on an Illumina HiSeq 2000 (Illumina, San Diego, CA, USA), and qRT-PCR was used to examine the relative abundance of m^6^A SAV1 RNA. The sequences of the SAV1 primers were 5′-ATGCTGTCCCGAAAGAAAACC-3′ and 5′-AGGCATAAGATTCCGAAGCAGA-3′.

### MeRIP-sequencing Data analysis

The exomePeak (Version 3.8) software was used for peak calling. The m6A peaks were annotated using bedtools (Version 2.25.0). The deepTools (version 2.4.1) was used for peak distribution analysis. The differentially m6A peaks were identified by a python script, using fisher test. Sequence motifs enriched in m6A peak regions were identified using Homer (version 4.10). Gene ontology (GO) analysis and Kyoto encyclopedia of genes and genomes (KEGG) enrichment analysis for annotated genes were both implemented by KOBAS software (version: 2.1.1).

### Statistical analysis

The difference between groups was analysed as by Student's t-test, and multiple groups were analysed when applicable by one-way ANOVA in Prism 7.0 (GraphPad Software, San Diego, CA, USA). Experiments were carried out in triplicate. A P-value <0.05 was considered statistically significant.

## Results

### Elevated expression of ALKBH5 in MM patients and MM cell lines

**Figure 1:**
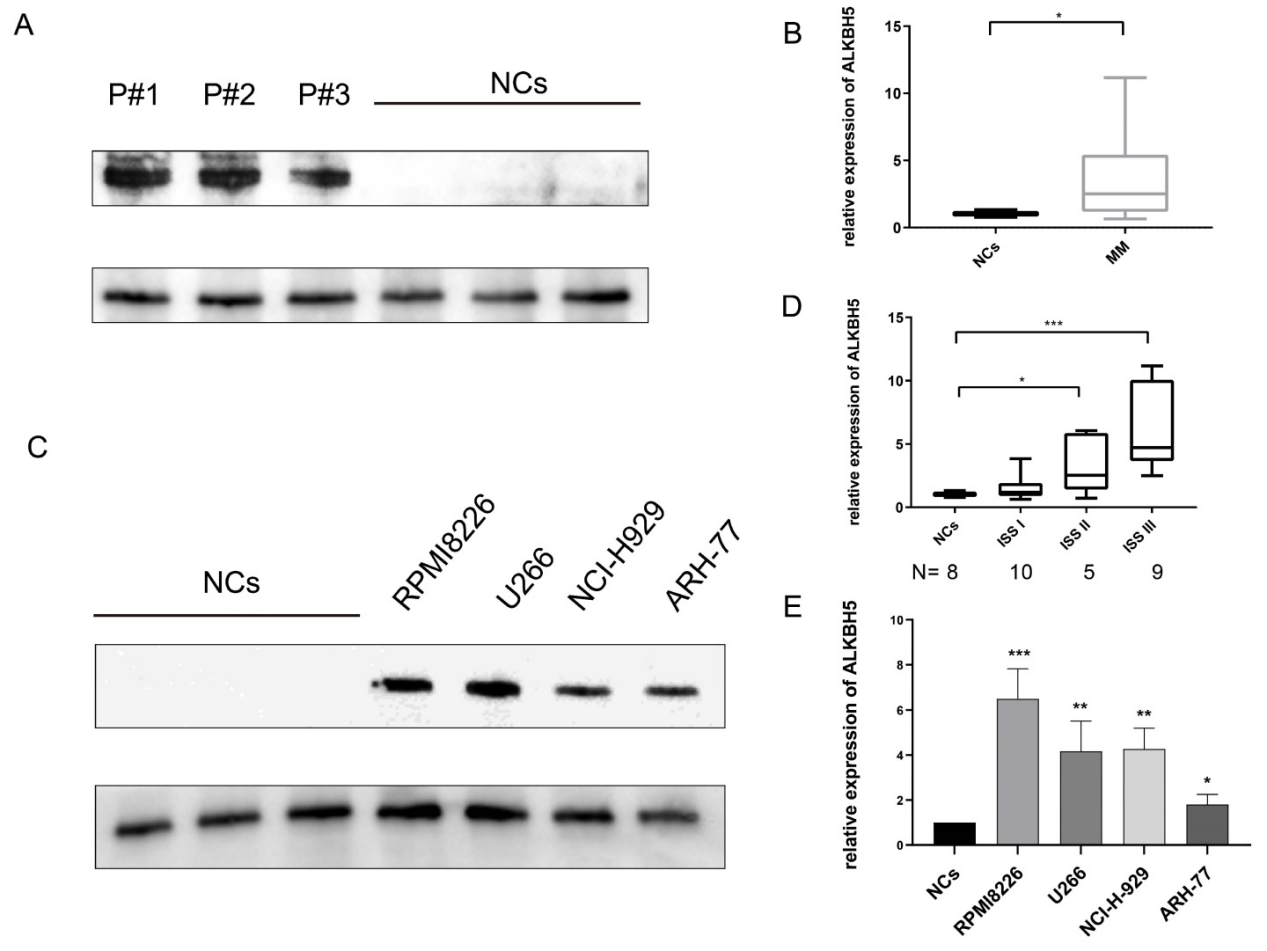
** Elevated ALKBH5 level in MM patient-derived CD138^+^ cells and MM cell lines.** (A) The expression level of ALKBH5 in primary CD138^+^ cells from MM patients (n=15) and normal volunteers (NCs, n=15) were detected by western blot. Each band represents 5 patients as samples were randomly divided into 3 groups to get enough protein. (B) The expression level of ALKBH5 in CD138^+^ cells from MM patients (n=25) and normal volunteers (NCs, n=25) were detected by qRT-PCR. (C) The expression level of ALKBH5 in MM cell lines (RPMI8226, ARH-77, NCI-H929 and U266 cells) were examined by western blot. (D) The expression level of ALKBH5 in different stages of MM patients was compared by qRT-PCR. The images shown are representative of three independent experiments. (E) The expression level of ALKBH5 in MM cell lines (RPMI8226, ARH-77, NCI-H929 and U266 cells) were examined through qRT-PCR. *P<0.5, **P<0.01, ***P<0.001.

We first investigated the expression of ALKBH5 in bone marrow-derived CD138^+^ cells from 8 healthy volunteers (normal control, NC) and 24 newly diagnosed MM patients through qRT-PCR. Then we verified the levels of ALKBH5 in 9 healthy volunteers and 25 newly diagnosed MM patients by western blot. The results showed that the expression of ALKBH5 in the CD138^+^ MM cells was significantly higher than that in the NC cells (Figure 1A and 1B). We also found that the ALKBH5 mRNA level was positively correlated with the MM stage according to the International Staging System (ISS) (Figure 1D). Consistently, the expression of ALKBH5 in myeloma cell lines, including RPMI8226, ARH-77, NCI-H929 and U266 cells, was more abundant than that in the normal controls (P<0.05) (Figure 1C and 1E). Collectively, these data suggested that ALKBH5 was highly expressed in both the MM patients and MM cell lines.

### Effect of ALKBH5 on myeloma cell proliferation, apoptosis, neovascularization, migration and invasion *in vitro*

**Figure 2:**
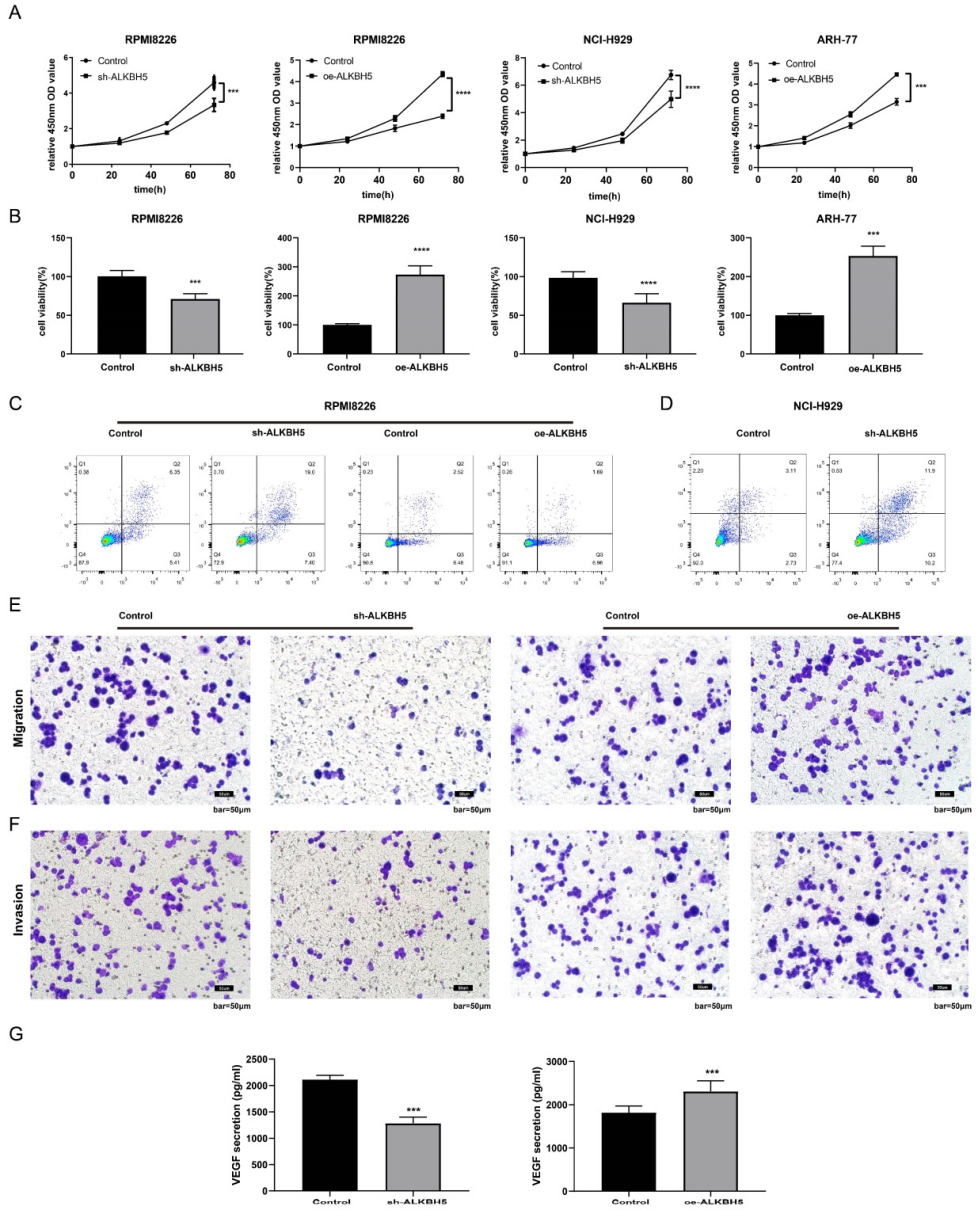
** Effect of ALKBH5 on myeloma cell proliferation, apoptosis, angiogenesis, migration, and invasion *in vitro*.** (A) and (B) The proliferation and viability of ALKBH5^-^ RPMI8226, ALKBH5^-^ NCI-H929, ALKBH5^+^ RPMI8226, ALKBH5^+^ ARH77 cells and control cells were assessed by CCK-8 assays. Annexin V-PE/7AAD staining was performed to examine apoptosis rate of the ALKBH5^+^ and ALKBH5^-^ RPMI8226 cells(C) and NCI-H929 cells(D), (E) and (F) The invasion and migration abilities of ALKBH5^-^ and ALKBH5^+^ RPMI8226 myeloma cells were assessed by transwell assays.(G) The VEGF levels of ALKBH5^-^ and ALKBH5^+^ RPMI8226 myeloma cells were assessed by ELISA. Data are expressed as the mean ± SEM of each group from three separate experiments. ***P<0.001, ****P<0.0001.

To characterize the role of ALKBH5 in the pathogenesis of MM, ALKBH5-RNAi, ALKBH5-OE and control lentiviruses containing GFP were transfected into RPMI8226 cells. The transfection efficiency was confirmed by fluorescence microscopy, qRT-PCR and western blot. The expression level of ALKBH5 in the RPMI8226 cells transfected with ALKBH5-RNAi (ALKBH5^-^ cells) or ALKBH5-OE (ALKBH5^+^ cells) lentivirus were significantly downregulated and upregulated, respectively, compared with the control cells (see Figure S1). Subsequently, we evaluated the proliferation of myeloma cells by CCK-8 assay and found that the proliferation of the ALKBH5^-^ RPMI8226 cells was prominently inhibited in contrast to the control cells (P<0.0001, Figure 2A). Similarly, the downregulation of ALKBH5 also suppressed the viability of the NCI-H929 cells (P<0.001, Figure 2B). The overexpression of ALKBH5 promoted the proliferation of RPMI8226 and ARH-77 cells (P<0.001, Figure 2A and 2B). Flow cytometry analysis showed that ALKBH5 deficiency increased the apoptosis rate of the RPMI8226 cells and NCI-H929 cells (P<0.05), while upregulation of ALKBH5 did not lead to a significant change (Figure 2C&D and figure S2). What's more, transwell assays were used to examine cell migration and invasion ability in different group. Our results indicated that cell migration and invasion ability were significantly downregulated in the ALKBH5^-^ group and upregulated in the ALKBH5^+^ group (P<0.05; Figure 2E-2F). Additionally, the vascular endothelial growth factor (VEGF), as a core proangiogenic cytokine, plays an important part in MM progress. So we tested the VEGF level in supernatant of cultured ALKBH5^-^ cells and ALKBH5^+^ cells through VEGF ELISA kit, respectively. It showed that knockdown of ALKBH5 could inhibit the VEGF secretion ability in myeloma cells (P< 0.001, Figure 2G).

### ALKBH5 inhibition exerts anti-myeloma effect *in vivo*

**Figure 3:**
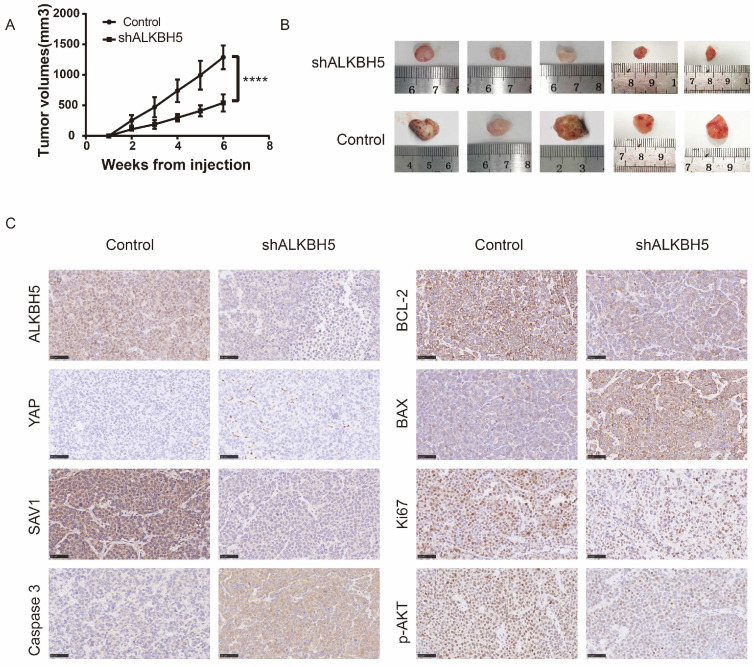
** ALKBH5 deficiency repressed the growth of MM cells *in vivo.*** (A) Tumour growth curves for the mice injected with ALKBH5^-^ and control RPMI8226 cells. (B) Images of the tumour sections from the NOD/SCID mouse xenograft models (ALKBH5^-^ : n=5, Control: n=5). (C) Representative immunohistochemical staining for ALKBH5, YAP, SAV1, Ki67, BAX, BCL-2, Pakt and caspase3 in the tumour tissues derived from mice injected with ALKBH5^-^ and control RPMI8226 cells. Bar=50μm. ****P<0.0001.

To verify the effect of ALKBH5 *in vivo*, we developed 6 xenograft models in NOD/SCIDs through subcutaneous injection of AKBH5^-^ and control RPMI8226 cells (ALKBH5^-^ : n=3, control: n=3). As expected, xenografts established with ALKBH5^-^ myeloma cells grew significantly slowlier than the controls (P<0.001, Figure 3A). The tumour volumes in the mice injected with the ALKBH5^-^ cells were obviously smaller compared to those in control mice (Figure 3B). Immunohistochemical staining for BCL-2, BAX and caspase-3 in tumour sections exhibited a marked decrease in BCL-2, Ki67 and pAKT and an increase in BAX and caspase-3 activity and expression in the tumours generated with ALKBH5^-^ cells (Figure 3C). Together, these results indicate that ALKBH5 inhibition has a protective effect on tumour growth *in vivo*.

### Identification of the target genes of ALKBH5 via MeRIP sequencing analysis

**Figure 4:**
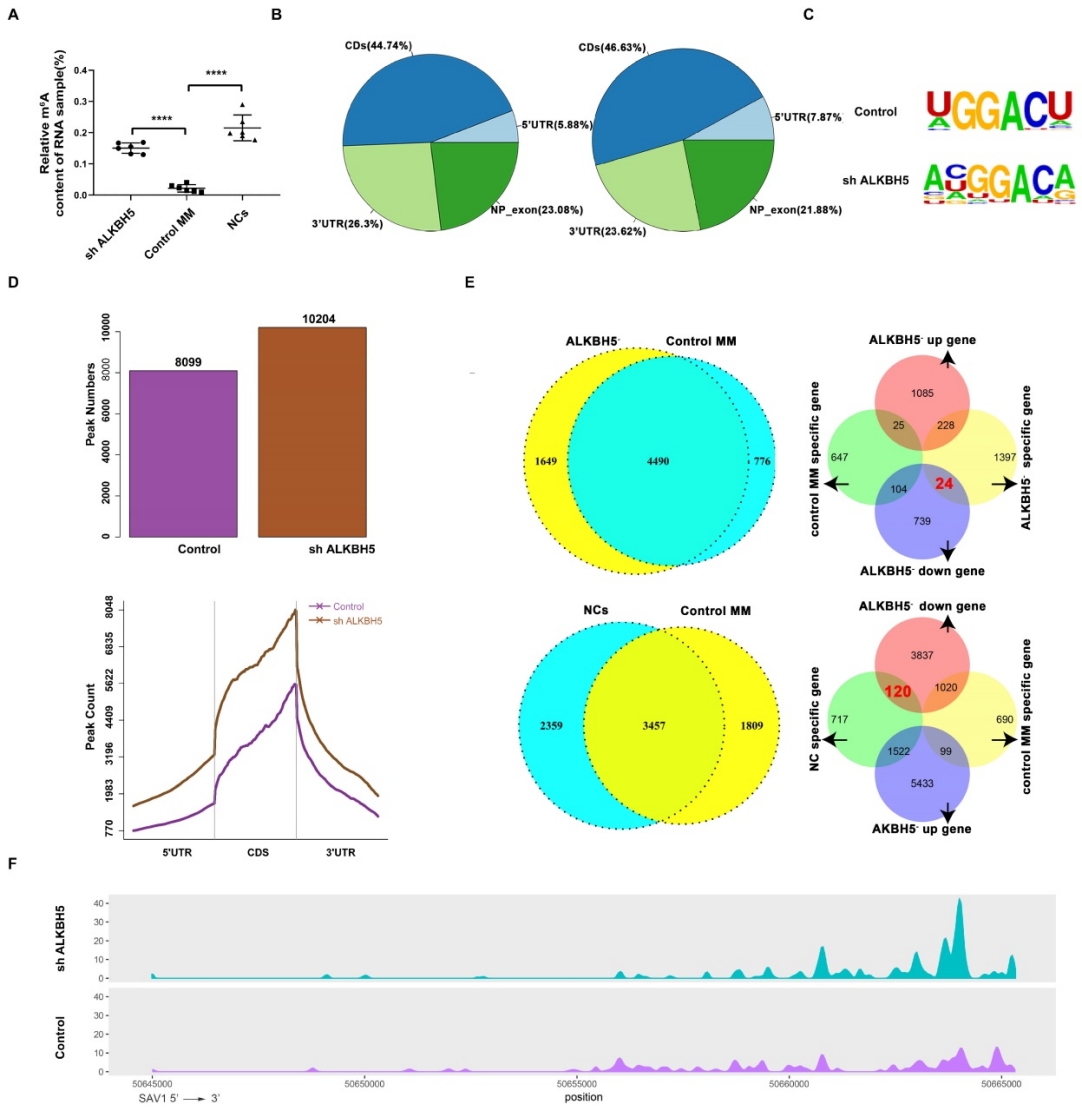
** Features of the m^6^A methylome and mRNA level changes in ALKBH5^-^ and control RPMI8226 cells.** (A) Relative m^6^A content of the RNA in the ALKBH5^-^, control RPMI8226 cells and the NCs. (B) Charts of the m6A peak distribution indicating the proportion of peaks in different regions of the ALKBH5^-^ and control RPMI8226 cells. (C) The m^6^A consensus motif in ALKBH5^-^ and control RPMI8226 cells was analysed by HOMER. (D) m^6^A peak counts representing the ALKBH5^-^ and control RPMI8226 cells were identified by MeRIP-seq. (E) The measurement of genes that changed in a m^6^A manner in ALKBH5^-^, control RPMI8226 cells and CD138^+^ cells from healthy volunteers (NCs). 24 genes were downregulated in 1649 ALKBH5^-^-specific genes and 120 genes were downregulated in NC-specific genes. (F) Tracks of m^6^A modifications of SAV1 mRNA in the ALKBH5^-^ and control RPMI8226 cells.

Firstly, the m^6^A level in the RNA samples taken from ALKBH5^-^ MM cells, control MM cells and bone marrow-derived CD138^+^ cells from healthy volunteers (normal controls, NCs) were detected using an EpiQuick m^6^A RNA methylation quantification kit and found that the m^6^A methylation levels in the NCs and ALKBH5^-^ myeloma cells were notably higher than those in the control MM cells (Figure 4A). To further investigate the variation of m^6^A modification in the transcripts, we mapped the transcriptome-wide m^6^A-methylomes in the ALKBH5^-^ and control MM cells as well as NCs by MeRIP-seq (methylated RNA immunoprecipitation with next-generation sequencing) as previously reported.^24^ According to our data, the m^6^A modifications were mainly located at the CDS, 3'UTR or stop codon regions (Figure 4B), and the consensus motif identified by HOMER was “GGACA(U)” (Figure 4C). A total of 10204 m^6^A peaks were detected in the ALKBH5^-^ cells, which was notably higher than that in the control cells (8044) (Figure 4D). Further analysis identified that 1649 transcripts were only identifid in ALKBH5^-^ group while not in control group, called “ALKBH5^-^-specific genes”. And m^6^A modifications were specifically found on these ALKBH5^-^-specific transcripts. Also, MeRIP-seq analysis showed that a total of 9786 m^6^A peaks were detected for the NC cells. Among them, 2359 transcripts has specific m^6^A peaks (only identifid in NCs group while not in control MM group) and were called “NC-specific genes”. Then, RNA sequencing analysis showed that 24 genes were downregulated in the “ALKBH5^-^-specific gene” group compared with control MM cells, and 120 genes were downregulated in the “NC-specific gene” group compared with control MM cells, and SAV1 gene was selected among the mutually downregulated genes in both groups (Figure 4E and 4F). SAV1 is an important component of the HIPPO pathway, which is highly conserved in mammals and plays a crucial role in the development of neoplasms and stem cell fate regulation. Therefore, we used SAV1 as the entry point to further explore the variations in other components of the HIPPO pathway.

### Knock-down of ALKBH5 suppressed myeloma progression via activating the Hippo pathway *in vivo and in vitro*

**Figure 5:**
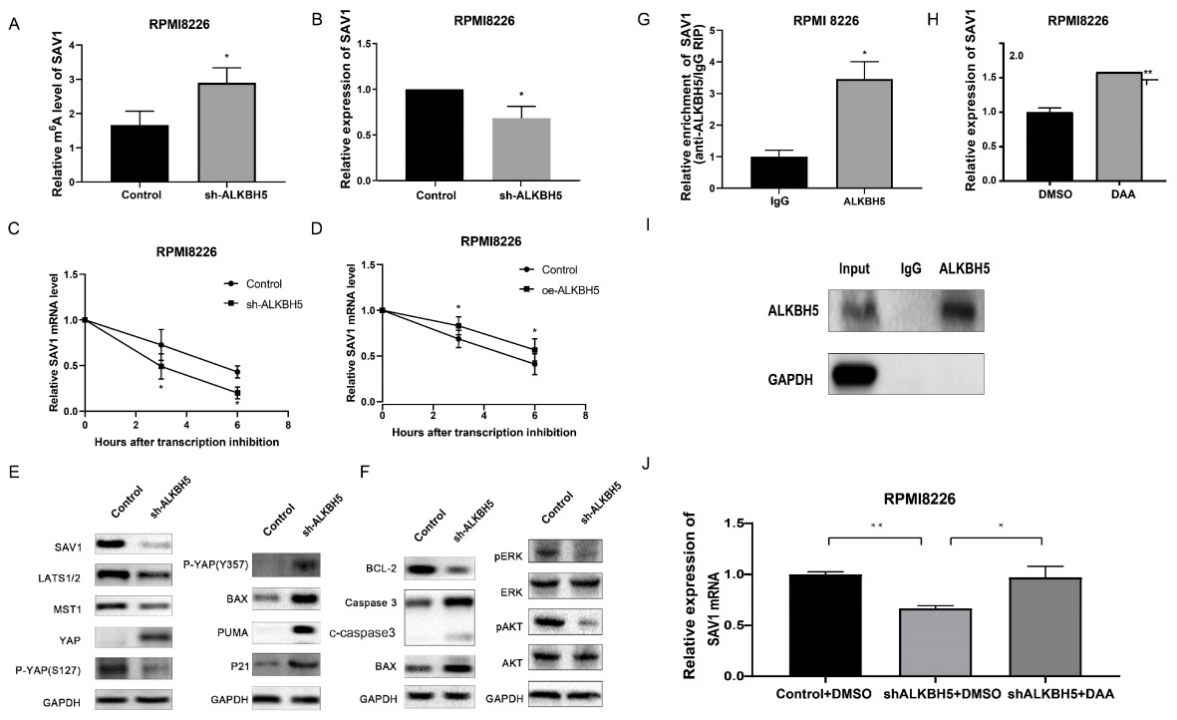
** Knockdown of ALKBH5 inhibited the activation of the HIPPO pathway.** (A) m^6^A enrichment of SAV1 was measured by m^6^A RIP-PCR in ALKBH5^-^ and control RPMI8226 cells. (B) qRT-PCR was used to measure SAV1 mRNA level in the ALKBH5^-^ and control RPMI8226 cells. (C) and (D) mRNA stability of SAV1 in the ALKBH5^+^ and ALKBH5^-^ RPMI8226 cells was detected by qRT-PCR. (E) Western blot revealed the expression of HIPPO pathway-related proteins, P-YAP (Y357) and P73-target genes (PUMA, P21 and BAX). (F) Western blot revealed the expression of proteins associated with myeloma cell proliferation and apoptosis. (G) RNA immunoprecipitation (RIP) assays in MM RPMI-8226 cells using ALKBH5 and IgG antibody. The ALKBH5-enriched SAV1 mRNA relative to the IgG-enriched value was calculated by qRT-PCR.(I)SAV1 mRNA expression was upregulated after demethylation treatment with DAA in MM RPMI-8226 cells. (H) and (J) Silencing ALKBH5 decreased SAV1 mRNA expression, while DAA treatment increased SAV1 mRNA expression after silencing ALKBH5 in MM cells. Data were mean ± SD. *P<0.5, **** P<0.0001.

First, we used gene-specific m^6^A qPCR to confirm the effect of ALKBH5 on SAV1 m^6^A level, and the m^6^A level of SAV1 mRNA was found to be increased in cells transfected with ALKBH5-RNAi lentivirus (Figure 5A). Moreover, qRT-PCR and western blot analyses confirmed that SAV1 expression was significantly decreased in the ALKBH5^-^ group (P<0.05, Figure 5B and 5E). Given that m^6^A modification in mRNA can regulate mRNA stability,^6^ an RNA stability assay was carried out to detect the changes of SAV1 mRNA stability, and the results showed that the SAV1 mRNA half-life was decreased after ALKBH5 was silenced (Figure 5C) and increased after ALKBH5 was overexpressed (Figure 5D).

In conclusion, ALKBH5 deficiency can regulate SAV1 mRNA stability and protein expression through m^6^A demethylation modification. Since SAV1 is the initiating protein of HIPPO pathway, other HIPPO pathway downstream proteins, including LATS1, MST1 and P-YAP (S127), were also detected and showed similar downward trends when ALKBH5 expression was knocked down (P<0.05, Figure 5E). The YAP protein, which was repressed by the activation of the HIPPO pathway, exhibited increased expression in the ALKBH5^-^ cells (P<0.05, Figure 5E). YAP can be bound by ABL1 in the cell nucleus and then can be phosphorylated on tyrosine 357, which augments the stability of P73, activates the proapoptotic gene and ultimately promotes the apoptosis of MM cells.^25^ Therefore, we further examined the levels of P73-target genes (P21, PUMA and BAX) and P-YAP (Y357) through western blot analysis. Consistent with our hypothesis, ALKBH5 knockdown substantially enhanced the P-YAP (Y357), P21, PUMA and BAX protein levels (P<0.05, Figure 5E). Accordingly, the levels of proteins associated with apoptosis, including BAX, BCL-2 and cleaved caspase3 as well as pERK and pAKT, showed a significant decline in the ALKBH5^-^ cells (P<0.05, Figure 5F). Consistently, immunohistochemical staining for SAV1 showed a marked decrease and YAP an increase in the xenograft generated with ALKBH5^-^ cells compared with control cells (Figure 3C). Additionally, RIP-qPCR further substantiated the direct binding between ALKBH5 and SAV1 mRNA (Figure 5G&I). To further determined whether ALKBH5 modulated SAV1 expression through its enzymatic activity, we used the drug 3-deazaadenosine (DAA) which suppress m6A methylation chemically.^26^ The results showed that the mRNA expression levels of SAV1 were significantly increased after DAA treatments in MM cells (Figure 5H). And the reduction of SAV1 mRNA caused by ALKBH5 knockdown was rescued by DAA treatment (Figure 5J).

Taken together, ALKBH5 inhibition could increase SAV1 m^6^A levels, suppressed the HIPPO signalling pathway and ultimately activates the transcription factor YAP in myeloma cells, promoted the expression of P21, PUMA and BAX, exerting an anti-myeloma effect* in vivo and in vitro*.

### ALKBH5 promotes myelomagenesis by up-regulating SAV1

**Figure 6:**
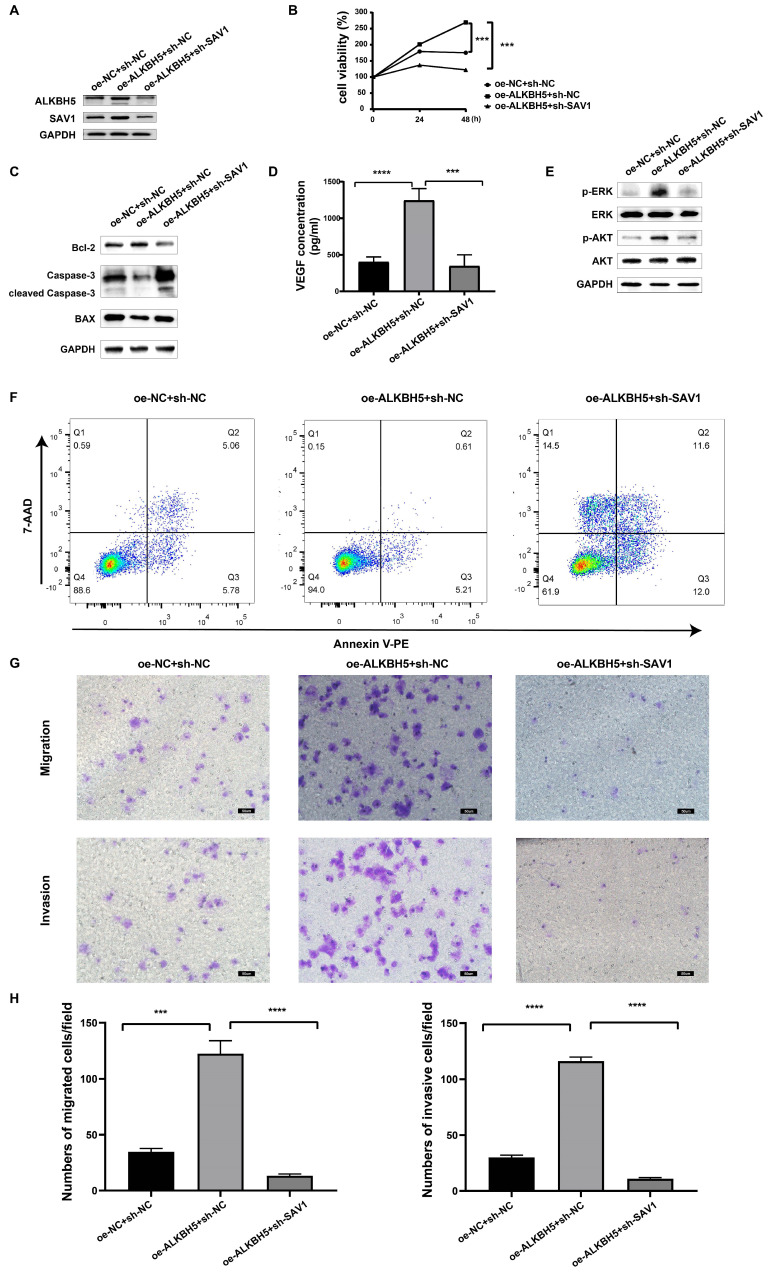
** SAV1 is involved in the process of ALKBH5-mediated myelomagenesis.** (A) SAV1 and ALKBH5 protein in oe-ALKBH5+sh-SAV1, oe-ALKBH5+shNC, and oe-NC+shNC RPMI 8226 cells. (B) cell viability in oe-ALKBH5+sh-SAV1 group, oe-ALKBH5+shNC group, and oe-NC+shNC group. (C) and (E) Western blot assays verified that ALKBH5 regulated apoptosis- and proliferation- related proteins synergized with SAV1. (D) The VEGF levels of oe-ALKBH5+sh-SAV1, oe-ALKBH5+shNC, and oe-NC+shNC myeloma cells were assessed by ELISA. (F) Annexin V-PE/7AAD staining was performed to examine apoptosis rate of oe-ALKBH5+sh-SAV1 group, oe-ALKBH5+shNC group, and oe-NC+shNC group. (G) and (H) The invasion and migration abilities of oe-ALKBH5+sh-SAV1, oe-ALKBH5+shNC, and oe-NC+shNC RPMI 8226 cells were assessed by transwell assays. Bar=50μm.***P<0.001, **** P<0.0001.

To determine the function of SAV1 in MM, rescue experiments were performed to identify whether SAV1 is involved in the process of ALKBH5-mediated myelomagenesis. We transfected sh-SAV1 lentivirus and control lentivirus into the ALKBH5-overexpressing MM cells, marked as oe-ALKBH5+sh-SAV1 group, oe-ALKBH5+shNC group, and oe-NC+shNC group. Western blot results displayed that the inhibition of SAV1 partially neutralized the effect of overexpressed ALKBH5 on both ALKBH5 and SAV1 protein expression in MM RPMI-8226 cells (Figure 6A). Cell viability assays (Figure 6B), VEGF ELISA assays (Figure 6D), cell apoptosis assays (Figure 6F) and cell migration/invasion assays (Figure 6G&H) and showed that SAV1-silencing in ALKBH5 overexpressing MM cells significantly attenuated the ALKBH5-mediated promotion of MM progression. Western blot assays further verified that ALKBH5 regulated proliferation- and apoptosis-related proteins synergized with SAV1 (Figure 6C&E). The *in vivo* cell derived xenograft (CDX) models also demonstrated that tumors from oe-ALKBH5+sh-NC group had an increase in tumor growth and progression. While ALKBH5-overexpression and SAV1-silencing group showed significantly delayed tumor growth and progression (Figure S4). These results indicate that ALKBH5 promotes myelomagenesis by up-regulating SAV1.

### ALKBH5 inhibition reduced the proportion of MM stem cells *in vitro*

**Figure 7:**
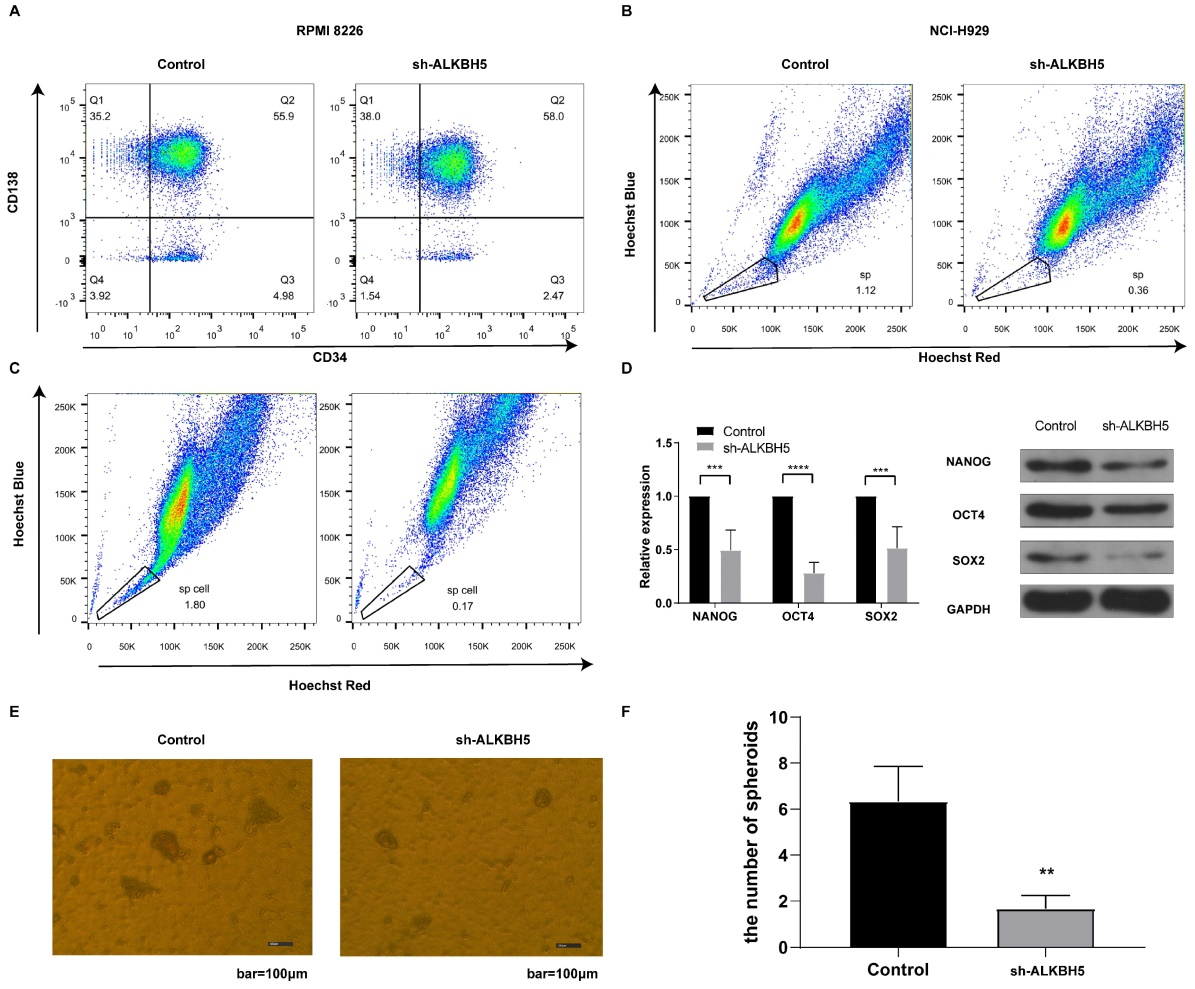
** ALKBH5 modulates the percentages of MM stem cells *in vitro.*** (A) The percentages of CD138^-^/CD34^-^ cells in the ALKBH5^-^ and control RPMI8226 cell populations were determined by flow cytometry. (B) Hoechst staining was used to show the variations in the percentages of side population cells in the ALKBH5^-^ and control NCI-H929 cell populations. (C) Hoechst staining was used to show the variations in the percentages of side population cells in the ALKBH5^-^ and control RPMI8226 cell populations. (D) NANOG, SOX2 and OCT4 mRNA and protein levels in the ALKBH5^-^ and control RPMI8226 cells were detected by qRT-PCR and western blot. (E) Representative images of stem cell spheroids for the ALKBH5^-^ and control cells. (F) Qualification of the number of stem cell spheroids in the ALKBH5^-^ and control cell populations. The data represent three independent replicates. ***P<0.001.

Since the HIPPO pathway is an essential component controlling stem cell self-renewal,^27^ and ALKBH5 has been suggested to promote the enrichment of breast cancer stem cells,^12^ we inferred that ALKBH5 might influence MM stem cell (MMSC) maintenance, proliferation, and survival. The phenotype of the MMSCs remains controversial because of their heterogeneity, and several markers, such as side population (SP) cells, CD138^-^/CD34^-^ cells and stem cell core genes, are most commonly used to identify MMSCs.^25^, ^28^-^30^ Therefore, we first detected the percentage of CD138^-^/CD34^-^ cells through flow cytometry. As expected, the ALKBH5^-^ group had fewer CD138^-^/CD34^-^ cells than the control group (P<0.05, Figure 7A). Furthermore, we also applied Hoechst staining to examine the percentage of SP cells in the RPMI8226 and NCI-H929 MM cell population via flow cytometry. Our results indicated that the percentage of SP cells in both kinds of ALKBH5^-^ myeloma cells were significantly lower than that in the corresponding control cells (P<0.05, Figure 7B and 7C). In addition, qRT-PCR and western blot were performed to detect the expression of NANOG, SOX2 and OCT4, which are genes associated with stem characteristics. The results showed that the levels of NANOG, SOX2 and OCT4 were notably decreased in the ALKBH5^-^ cells compared with those in the control cells (P<0.05, Figure 7D). Moreover, we assessed the sphere formation ability of the stem cells in ALKBH5- and control RPMI8226 cell populations and found that the number of stem cell spheres was decreased after ALKBH5 was downregulated (Figure 7E and 7F). Thus, our findings support the hypothesis that ALKBH5 deficiency inhibited the growth of MM cells by decreasing the percentage of MM stem cells.

## Discussion

In recent years, emerging evidence has indicated that to a large extent tumours rely on epigenetic regulation, including abnormal DNA methylation, histone methylation and acetylation modification, to escape immune surveillance and to develop drug resistance.^31^ However, epigenetic modification-targeted drugs, including hypomethylating agents and histone deacetylase inhibitors, have limited therapeutic effects in MM patients.^32^ These outcomes suggest that a new epigenetic molecular mechanism underlying the pathogenesis of MM remains to be clarified. M^6^A is the most extensive reversible chemical modification on eukaryotic mRNA.^33^ Our experimental results showed that the expression of ALKBH5 in CD138^+^ cells derived from myeloma patients and MM cell lines was significantly higher than that in normal control cells, and its expression level was positively correlated with the ISS stage of MM patients. Knockdown of the ALKBH5 gene inhibited myeloma cell proliferation, neovascularization, invasion and migration ability and promoted apoptosis both *in vitro* and *in vivo*. More importantly, we found that ALKBH5 deficiency in MM cells enhanced the RNA methylation of the HIPPO pathway-initiating protein SAV1, suppressed the activation of the HIPPO pathway, and ultimately reduced the proportion of myeloma stem cells. Conclusively, our data indicated that ALKBH5 can be a novel prognostic biomarker and a therapeutic target for MM.

It has been reported that ALKBH5 participates in tumorigenesis. However, the biological function of ALKBH5 is heterogeneous in various tumours. Most studies have demonstrated that ALKBH5 promotes tumorigenesis. The expression level of the ALKBH5 gene is positively correlated with the stage of ovarian cancer, and it can inhibit the autophagy of ovarian cancer through the mTOR pathway to promote the proliferation and invasion of ovarian cancer cells.^34^ Furthermore, a high level of ALKBH5 is an adverse prognostic factors in patients with malignant glioma. ALKBH5 can stabilize the expression of FOXM1 mRNA by eliminating the m^6^A modification of FOXM1 precursor RNA and enhance the self-renewal ability of glioma stem cells, thereby promoting the progression of malignant glioma.^35^ However, ALKBH5 inhibited pancreatic cancer motility and was positively associated with the prognosis of pancreatic cancer patients by reducing the methylation of the kcnk15-as1 lncRNA.^36^ Our research supported the supposition that ALKBH5 plays a carcinogenic role in MM progression, which is consistent with most studies. This verification suggested that ALKBH5 can be developed as a valuable therapeutic strategy for MM treatment.

The HIPPO pathway proteins MST1, MST2, LATS1, LATS2 and YAP/TAZ are regulated by each other and function as a complex network.^16^ Generally, the activation of SAV1 leads to the formation of the MST1/MST2 dimer, initiates a phosphorylation cascade and ultimately phosphorylates the transcriptional active factor YAP/TAZ which leads to its degradation and deactivation, finally resulting in the activation or deactivation of various transcription factors involved in cell proliferation, organ size, tissue regeneration and metastasis. YAP and TAZ are activated and translocated into the nucleus to promote cell proliferation in various tumours, including liver, breast, lung, colon, ovary, and others.^37^ More importantly, YAP has also been demonstrated to be a major inducer of cancer stem cell properties in osteosarcoma and glioblastoma.^38^ However, the initiation step of the HIPPO pathway remains elusive, which largely limits its clinical application. In the majority of cancers, the overall genetic mutation rate of Hippo pathway components is relatively low, and high YAP/TAZ activity is not correlated with genetic mutations of the Hippo pathway.^39^ Our work first demonstrated that, in MM, the abnormality of the HIPPO pathway-initiating protein SAV1 is not attributed to genetic mutations but to RNA methylation modification. ALKBH5 deficiency in myeloma cells significantly enhanced the SAV1 mRNA methylation level and therefore regulated its stability and expression, led to YAP phosphorylation and finally resulted in YAP deactivation.

The vascular endothelial growth factor (VEGF), as a core proangiogenic cytokine, plays an important part in MM progress. Bevacizumab, a monoclonal antibody targeting all human VEGF isoforms, has been testified to improve survival in most cancer patients.^40^ However, in MM, the addition of bevacizumab to anti-MM therapies (bortezomib or thalidomide) did not result in a significant improvement in the outcome of patients, which were probably related to the complexity of the BM microenvironment by a myriad of proangiogenic cytokines and other active pro-angiogenic pathways.^41^ Some latest reports displayed that m6A modifiers manipulated angiogenesis via modulating VEGF expression either directly targeting specific m6A sites of VEGF mRNA 42-44 or indirectly mediating by other downstream miRNAs and proteins. 45, 46 In our study, the secretion of VEGF decreased after silencing ALKBH5 gene expression in MM cells, addressing the angiogenesis-promoting effect of ALKBH5. Further rescue experiments displayed that ALKBH5 regulated the angiogenesis-promoting effect through SAV1. All these results indicate the potential possibility of an alternative or co-targeted therapy.

In recent years, emerging evidence has revealed that m^6^A methylation is involved in cancer stem cell generation and maintenance, governing cancer progression and therapeutic resistance, which highlight the potential of m^6^A methylation as a novel target for cancer therapeutics.^47^ ALKBH5 overexpression increased the percentage of breast cancer stem cell via inducing m^6^A -demethylation of NANOG mRNA. Furthermore, m^6^A reader YTHDF2 was shown to promote the liver cancer stem cell phenotype and cancer metastasis by regulating OCT4 expression via m^6^A RNA methylation.^48^ However, attributed to the ambiguous cancer stem cell marker and the difficulties in the isolation and culture of cancer stem cell, most studies only investigated the effect of RNA methylation on cancer stem cell phenotype. Only in glioblastoma stem-like cells (GSCs) and leukemia stem cells which have relative unequivocal marker, ALKBH5 was proved to be highly expressed in purified cancer stem cells. Silencing ALKBH5 suppresses the proliferation of patient-derived GSCs.^35^ Furthermore, ALKBH5 is found to be required for the development and maintenance of AML and self-renewal of leukemia stem/initiating cells.^13^ Similarly, precise stem cell surface marker is lacked in multiple myeloma. Therefore, in this study, we only demonstrated that ALKBH5 increased the percentage of SP cells and CD138^-^/CD34^-^ myeloma stem cells by activating cancer stem cell related HIPPO pathway signalling and promoting the expression of pluripotency factors NANOG, SOX2 and OCT4. These data suggest that ALKBH5 plays critical roles in MM stem cell maintenance. In the future, we need to address the function of ALKBH5 in purified myeloma stem cells which help its application as effective cancer stem cell targeted therapeutics.

## Supplementary Material

Supplementary figures and table.

## Abbreviations

MM: mutiple myeloma
ALKBH5: alkB homolog 5
M^6^A: N6-Methyladenosine
MeRIP-seq: methylated RNA immunoprecipitation with next generation sequencing
FTO: fat mass and obesity associated protein
METTL3: the methyltransferase like 3
METTL14: the methyltransferase like 14
YAP: yes-associated protein
SAV1: salvador family WW domain containing protein
NOD/SCID mice: nonobese diabetic/severe combined immunodeficienr mice

## References

[1] Anderson KC, Carrasco RD (2011). Pathogenesis of myeloma. Annu Rev Pathol.

[2] Durer C, Durer S, Lee S, Chakraborty R, Malik MN, Rafae A (2020). Treatment of relapsed multiple myeloma: Evidence-based recommendations. Blood Rev.

[3] Liu N, Parisien M, Dai Q, Zheng G, He C, Pan T (2013). Probing N6-methyladenosine RNA modification status at single nucleotide resolution in mRNA and long noncoding RNA. RNA.

[4] Alarcon CR, Lee H, Goodarzi H, Halberg N, Tavazoie SF (2015). N6-methyladenosine marks primary microRNAs for processing. Nature.

[5] Wang X, Zhao BS, Roundtree IA, Lu Z, Han D, Ma H (2015). N(6)-methyladenosine Modulates Messenger RNA Translation Efficiency. Cell.

[6] Wang X, Lu Z, Gomez A, Hon GC, Yue Y, Han D (2014). N6-methyladenosine-dependent regulation of messenger RNA stability. Nature.

[7] Ping XL, Sun BF, Wang L, Xiao W, Yang X, Wang WJ (2014). Mammalian WTAP is a regulatory subunit of the RNA N6-methyladenosine methyltransferase. Cell Res.

[8] Jia G, Fu Y, Zhao X, Dai Q, Zheng G, Yang Y (2011). N6-methyladenosine in nuclear RNA is a major substrate of the obesity-associated FTO. Nat Chem Biol.

[9] Meyer KD, Jaffrey SR (2017). Rethinking m(6)A Readers, Writers, and Erasers. Annu Rev Cell Dev Biol.

[10] Zheng G, Dahl JA, Niu Y, Fedorcsak P, Huang CM, Li CJ (2013). ALKBH5 is a mammalian RNA demethylase that impacts RNA metabolism and mouse fertility. Mol Cell.

[11] Tang B, Yang Y, Kang M, Wang Y, Wang Y, Bi Y (2020). m(6)A demethylase ALKBH5 inhibits pancreatic cancer tumorigenesis by decreasing WIF-1 RNA methylation and mediating Wnt signaling. Mol Cancer.

[12] Zhang C, Samanta D, Lu H, Bullen JW, Zhang H, Chen I (2016). Hypoxia induces the breast cancer stem cell phenotype by HIF-dependent and ALKBH5-mediated m(6)A-demethylation of NANOG mRNA. Proc Natl Acad Sci U S A.

[13] Shen C, Sheng Y, Zhu AC, Robinson S, Jiang X, Dong L (2020). RNA Demethylase ALKBH5 Selectively Promotes Tumorigenesis and Cancer Stem Cell Self-Renewal in Acute Myeloid Leukemia. Cell Stem Cell.

[14] Kumar S, Nagpal R, Kumar A, Ashraf MU, Bae YS (2021). Immunotherapeutic Potential of m6A-Modifiers and MicroRNAs in Controlling Acute Myeloid Leukaemia. Biomedicines.

[15] Misra JR, Irvine KD (2018). The Hippo Signaling Network and Its Biological Functions. Annu Rev Genet.

[16] Yu FX, Zhao B, Guan KL (2015). Hippo Pathway in Organ Size Control, Tissue Homeostasis, and Cancer. Cell.

[17] Jeong SH, Kim HB, Kim MC, Lee JM, Lee JH, Kim JH (2018). Hippo-mediated suppression of IRS2/AKT signaling prevents hepatic steatosis and liver cancer. J Clin Invest.

[18] Lee WY, Chen PC, Wu WS, Wu HC, Lan CH, Huang YH (2017). Panobinostat sensitizes KRAS-mutant non-small-cell lung cancer to gefitinib by targeting TAZ. Int J Cancer.

[19] Vici P, Ercolani C, Di Benedetto A, Pizzuti L, Di Lauro L, Sperati F (2016). Topographic expression of the Hippo transducers TAZ and YAP in triple-negative breast cancer treated with neoadjuvant chemotherapy. J Exp Clin Cancer Res.

[20] Wang L, Shi S, Guo Z, Zhang X, Han S, Yang A (2013). Overexpression of YAP and TAZ is an independent predictor of prognosis in colorectal cancer and related to the proliferation and metastasis of colon cancer cells. PLoS One.

[21] Maruyama J, Inami K, Michishita F, Jiang X, Iwasa H, Nakagawa K (2018). Novel YAP1 Activator, Identified by Transcription-Based Functional Screen, Limits Multiple Myeloma Growth. Mol Cancer Res.

[22] Gagliardi M, Matarazzo MR (2016). RIP: RNA Immunoprecipitation. Methods Mol Biol.

[23] Matsui W, Huff CA, Wang Q, Malehorn MT, Barber J, Tanhehco Y (2004). Characterization of clonogenic multiple myeloma cells. Blood.

[24] Meyer KD, Saletore Y, Zumbo P, Elemento O, Mason CE, Jaffrey SR (2012). Comprehensive analysis of mRNA methylation reveals enrichment in 3' UTRs and near stop codons. Cell.

[25] Levy D, Adamovich Y, Reuven N, Shaul Y (2008). Yap1 phosphorylation by c-Abl is a critical step in selective activation of proapoptotic genes in response to DNA damage. Mol Cell.

[26] Fustin JM, Doi M, Yamaguchi Y, Hida H, Nishimura S, Yoshida M (2013). RNA-methylation-dependent RNA processing controls the speed of the circadian clock. Cell.

[27] Park JH, Shin JE, Park HW (2018). The Role of Hippo Pathway in Cancer Stem Cell Biology. Mol Cells.

[28] Du J, Liu S, He J, Liu X, Qu Y, Yan W (2015). MicroRNA-451 regulates stemness of side population cells via PI3K/Akt/mTOR signaling pathway in multiple myeloma. Oncotarget.

[29] Beyar-Katz O, Magidey K, Reiner-Benaim A, Barak N, Avivi I, Cohen Y (2019). Proinflammatory Macrophages Promote Multiple Myeloma Resistance to Bortezomib Therapy. Mol Cancer Res.

[30] Ai L, Mu S, Sun C, Fan F, Yan H, Qin Y (2019). Myeloid-derived suppressor cells endow stem-like qualities to multiple myeloma cells by inducing piRNA-823 expression and DNMT3B activation. Mol Cancer.

[31] Chiappinelli KB, Zahnow CA, Ahuja N, Baylin SB (2016). Combining Epigenetic and Immunotherapy to Combat Cancer. Cancer Res.

[32] Medical Masterclass c, Firth J (2019). Haematology: multiple myeloma. Clin Med (Lond).

[33] Yue Y, Liu J, He C (2015). RNA N6-methyladenosine methylation in post-transcriptional gene expression regulation. Genes Dev.

[34] Zhu H, Gan X, Jiang X, Diao S, Wu H, Hu J (2019). ALKBH5 inhibited autophagy of epithelial ovarian cancer through miR-7 and BCL-2. J Exp Clin Cancer Res.

[35] Zhang S, Zhao BS, Zhou A, Lin K, Zheng S, Lu Z (2017). m(6)A Demethylase ALKBH5 Maintains Tumorigenicity of Glioblastoma Stem-like Cells by Sustaining FOXM1 Expression and Cell Proliferation Program. Cancer Cell.

[36] He Y, Hu H, Wang Y, Yuan H, Lu Z, Wu P (2018). ALKBH5 Inhibits Pancreatic Cancer Motility by Decreasing Long Non-Coding RNA KCNK15-AS1 Methylation. Cell Physiol Biochem.

[37] Zanconato F, Cordenonsi M, Piccolo S (2016). YAP/TAZ at the Roots of Cancer. Cancer Cell.

[38] Basu-Roy U, Bayin NS, Rattanakorn K, Han E, Placantonakis DG, Mansukhani A (2015). Sox2 antagonizes the Hippo pathway to maintain stemness in cancer cells. Nat Commun.

[39] Yu T, Bachman J, Lai ZC (2013). Evidence for a tumor suppressor role for the large tumor suppressor genes LATS1 and LATS2 in human cancer. Genetics.

[40] Itatani Y, Kawada K, Yamamoto T, Sakai Y (2018). Resistance to Anti-Angiogenic Therapy in Cancer-Alterations to Anti-VEGF Pathway. Int J Mol Sci.

[41] Ria R, Melaccio A, Racanelli V, Vacca A (2020). Anti-VEGF Drugs in the Treatment of Multiple Myeloma Patients. J Clin Med.

[42] Jiang L, Li Y, He Y, Wei D, Yan L, Wen H (2021). Knockdown of m6A Reader IGF2BP3 Inhibited Hypoxia-Induced Cell Migration and Angiogenesis by Regulating Hypoxia Inducible Factor-1alpha in Stomach Cancer. Front Oncol.

[43] Yang Z, Wang T, Wu D, Min Z, Tan J, Yu B (2020). RNA N6-methyladenosine reader IGF2BP3 regulates cell cycle and angiogenesis in colon cancer. J Exp Clin Cancer Res.

[44] Wang G, Dai Y, Li K, Cheng M, Xiong G, Wang X (2021). Deficiency of Mettl3 in Bladder Cancer Stem Cells Inhibits Bladder Cancer Progression and Angiogenesis. Front Cell Dev Biol.

[45] Wang H, Deng Q, Lv Z, Ling Y, Hou X, Chen Z (2019). N6-methyladenosine induced miR-143-3p promotes the brain metastasis of lung cancer via regulation of VASH1. Mol Cancer.

[46] Xiao Y, Thakkar KN, Zhao H, Broughton J, Li Y, Seoane JA (2020). The m(6)A RNA demethylase FTO is a HIF-independent synthetic lethal partner with the VHL tumor suppressor. Proc Natl Acad Sci U S A.

[47] Ma Z, Ji J (2020). N6-methyladenosine (m6A) RNA modification in cancer stem cells. Stem Cells.

[48] Zhang C, Huang S, Zhuang H, Ruan S, Zhou Z, Huang K (2020). YTHDF2 promotes the liver cancer stem cell phenotype and cancer metastasis by regulating OCT4 expression via m6A RNA methylation. Oncogene.

