# Supplementary material for: ALKBH5 Promotes Multiple Myeloma Tumorigenicity through inducing m6A-demethylation of SAV1 mRNA and Myeloma Stem Cell Phenotype
Source: Int J Biol Sci. 2022 Mar 6;18(6):2235–48. doi: 10.7150/ijbs.64943 (PMC8990482; doi:10.7150/ijbs.64943)
Supplement: Supplementary file 1 — Supplementary figures and table. [file ijbsv18p2235s1.pdf]

Supplemental files

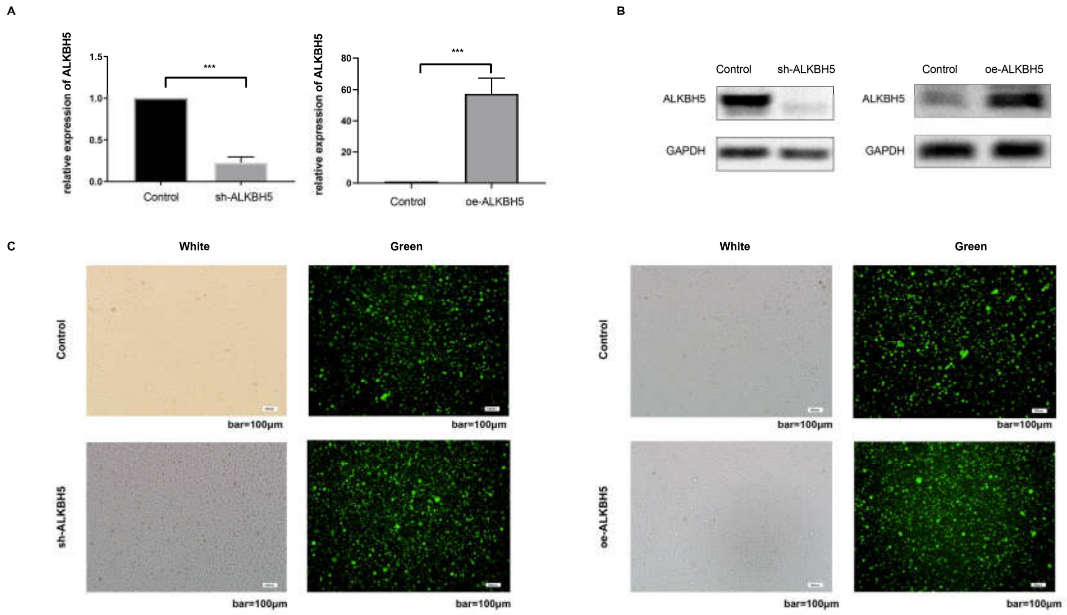

Figure S1. The transfection efficiency of lentivirus in RPMI8226 cells

(A) The ALKBH5 mRNA level in oe-ALKBH5 and sh-ALKBH5 RPMI8226 cells. (B) The expression levels of ALKBH5 in oe-ALKBH5 and sh-ALKBH5 myeloma cells. (C) Representative images of transfected RPMI8226 cells under fluorescence microscopy. White: general microscopy field, Green: fluorescence microscopy field, bar=100μm***P<0.001.

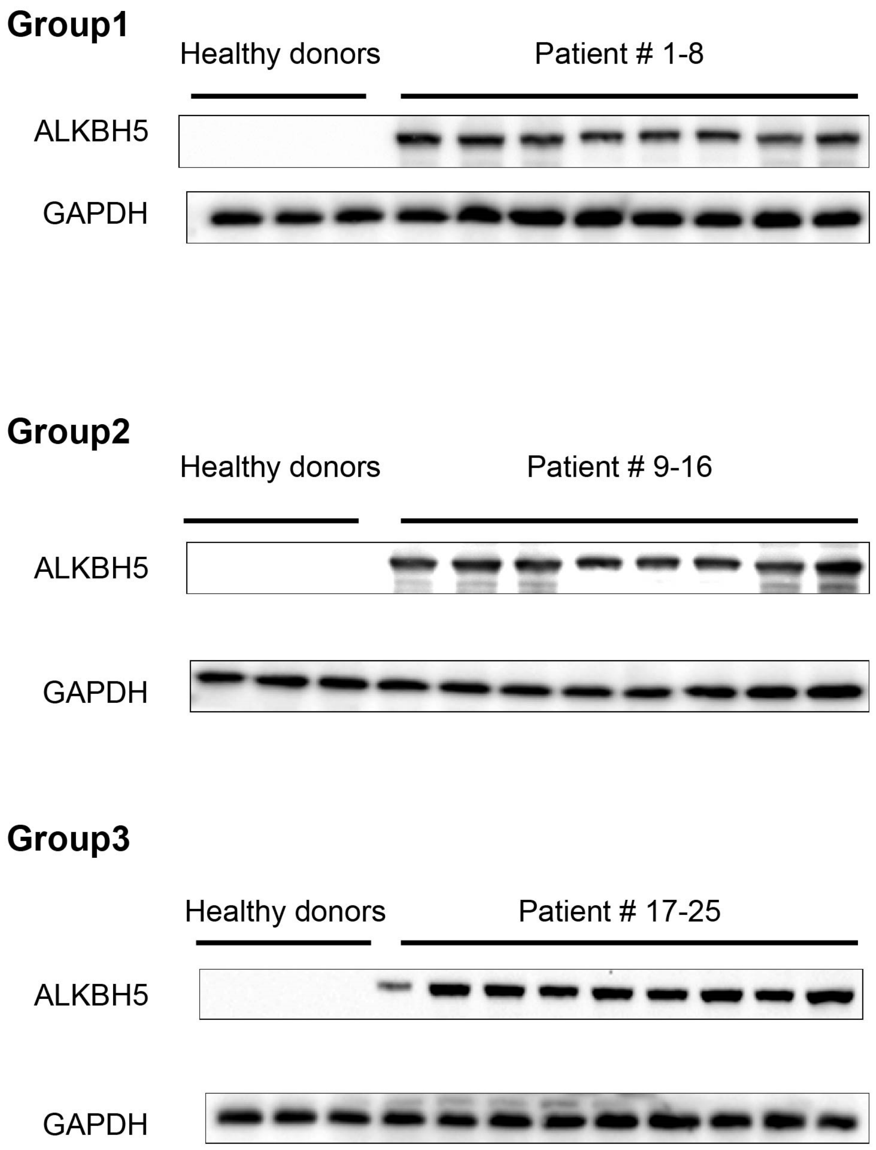

Figure S2. The expression of ALKBH5 from individual patient examined by western blot

Patient	Gender	Age (year)	MM stage (ISS)	LDH (U/L)	β2-MG (mg/L)	Serum Albumin (g/L)	MM-FISH
**1**	female	50	II	75	4.7	23.3	t(4;14)(p16;q32)
**2**	male	68	III	90	7.5	24.5	
**3**	female	71	I	139	1.9	39.6	
**4**	male	65	I	187	1.4	38.5	
**5**	female	69	I	89	3.2	36	t(4;14)(p16;q32)
**6**	female	57	III	305	17.1	38.6	t(4;14)(p16;q32)
**7**	male	57	I	149	1.2	43	
**8**	female	74	II	300	3.9	19	
**9**	female	73	III	325	13.1	27.7	t(4;14)(p16;q32)
**10**	female	63	III	225	59.3	21.4	
**11**	female	57	II	320	4.3	20	
**12**	female	71	I	197	2.2	36.7	
**13**	female	69	III	288	3.6	30.9	
**14**	female	69	I	99	3.1	37.8	
**15**	female	56	II	165	5.1	35	
**16**	male	59	III	340	7.5	18.6	
**17**	female	59	I	125	1.6	32.7	
**18**	female	71	I	150	2.5	41.2	
**19**	female	74	II	136	4.7	32.5	
**20**	male	81	III	129	8.5	35.1	
**21**	female	68	I	205	1.8	36.1	
**22**	female	69	III	166	7.6	32	t(11;14)(q13;q32)
**23**	female	64	I	254	2.8	39.5	
**24**	male	65	III	183	13	28.8	
**25**	male	66	III	220	5.6	17.9	

Table S1. The patients' information

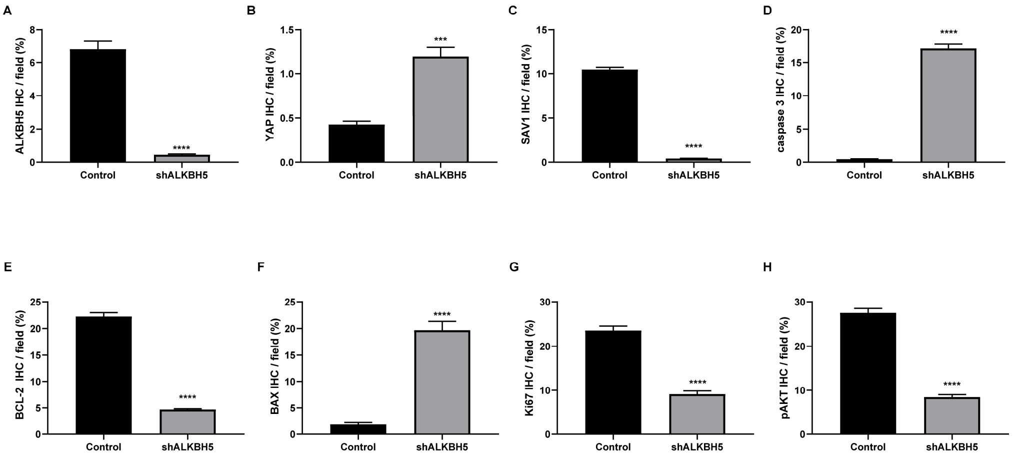

Figure S3. Stastical diaram of the IHC result of figure 3C

(A)-(F) Qualification of ALKBH5, YAP, SAV1, caspase 3, BCL-2, BAX, Ki67 and pAKT IHC result in the ALKBH5^{-} and control cell populations. ***P<0.001,****P<0.0001.

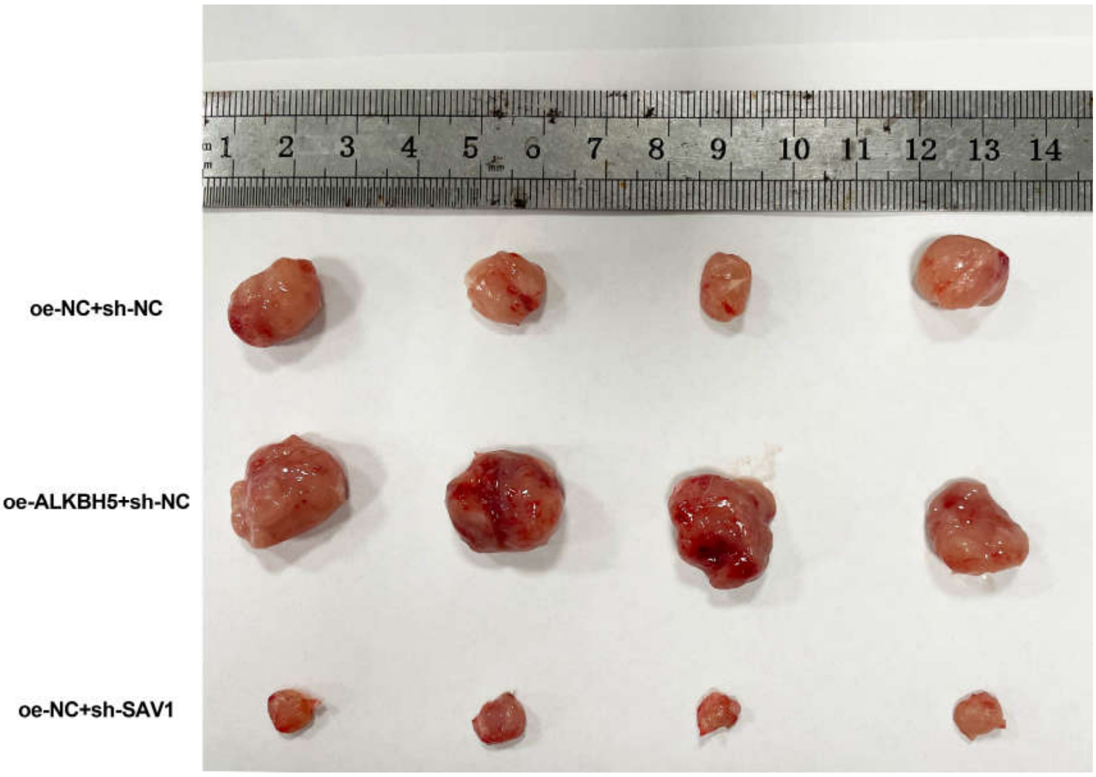

Figue S4. The in vivo cell derived xenograft models showed that tumors from oe-ALKBH5+sh-NC group had an increase in tumor volume and significantly delayed in ALKBH5-overexpression and SAV1-silencing group.
